# Supplementary material for: Miocene African topography induces decoupling of Somali Jet and South Asian summer monsoon rainfall
Source: Nat Commun. 2025 Aug 4;16:7172. doi: 10.1038/s41467-025-62186-y (PMC12322274; doi:10.1038/s41467-025-62186-y)
Supplement: Supplementary file 1 — Supplementary Information [file 41467_2025_62186_MOESM1_ESM.docx]

**Miocene African topography induces decoupling of Somali Jet and South Asian summer monsoon rainfall**

Zixuan Han^1,2,3^; Niklas Werner^2^; Zhenqian Wang^2^; Xiangyu Li^4,5^; Zhengquan Yao^6,7^;

Qiong Zhang^2*^

^1^Key Laboratory of Marine Hazards Forecasting, Ministry of Natural Resources, Hohai University, Nanjing, China

^2^Department of Physical Geography and Bolin Centre for Climate Research, Stockholm University, Stockholm, Sweden

^3^College of Oceanography, Hohai University, Nanjing, China

^4^Department of Atmospheric Science, School of Environmental Studies, China University of Geosciences, Wuhan, China

^5^Centre for Severe Weather and Climate and Hydro-geological Hazards, Wuhan, 430078, China

^6^Key Laboratory of Marine Geology and Metallogeny, First Institute of Oceanography, Ministry of Natural Resources, Qingdao, China

^7^Laboratory for Marine Geology, Qingdao Marine Science and Technology Center, Qingdao, China

**Corresponding authors:* Qiong Zhang [(qiong.zhang@natgeo.su.se)](mailto:(qiong.zhang@natgeo.su.se))

Contents of this file

Supplementary Table 1

Supplementary Figures 1 to 13

**Supplementary Table 1** **Summary of seven experiments with corresponding topography and *p*CO_2_ values using EC-Earth3 model**

| **Experiment** | **PI** | **MT05** | **MT15** | **MT25** | **MC05** | **MC15** | **MC25** |
| --- | --- | --- | --- | --- | --- | --- | --- |
| Topography | PI | MT05 | MT15 | MT25 | MT05 | MT15 | MT25 |
| *p*CO2 (p.p.m.v) | 284 | 284 | 284 | 284 | 500 | 500 | 500 |
| Vegetation | Dynamic | Dynamic | Dynamic | Dynamic | Dynamic | Dynamic | Dynamic |


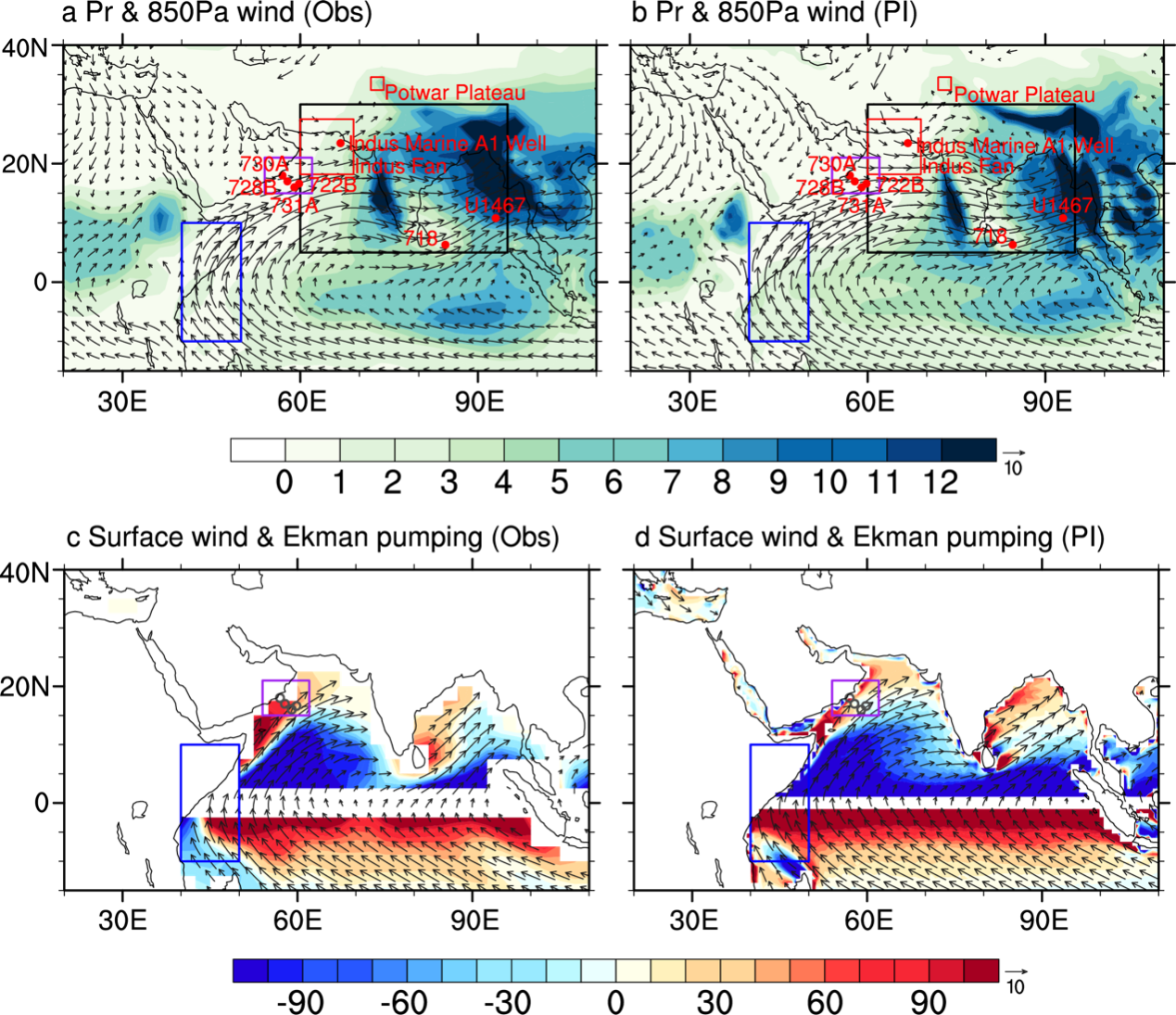


**Supplementary Figure 1** **Model validation.** **a** Observed climatological mean rainfall (mm day^-1^) and 850-hPa wind (m s^-1^), and **c** surface wind (vectors, m s^-1^) and its induced Ekman pumping (cm day^-1^) during boreal summer from 1980 to 2010. **b** and **d** are the same as **a** and **c**, but for the results of pre-industrial simulation from EC-Earth3 model. Monthly rainfall in **a** is from the Global Precipitation Climatology Project (GPCP)^1^, and 850-hPa and surface horizontal winds are from the National Centers for Environmental Prediction reanalysis 2 (NCEP2)^2^. In **a** and **b**, red dots or red boxes denote the locations of the proxy records in Figure 1**i**-**o**, and black boxes (5° N-30° N; 60° E-95° E) mark the SASM region. In **c** and **d**, gray circles in western Arabian Sea denote the locations of the proxy records (same as in Supplementary Figure 1**a** and **b**). In **a**-**d**, blue boxes (10º S-10º N, 40º -50º E) mark the cross-equatorial Somali Jet and purple boxes (15º -21º N, 54º -62º E) mark the western Arabian Sea upwelling region that proxy records located.


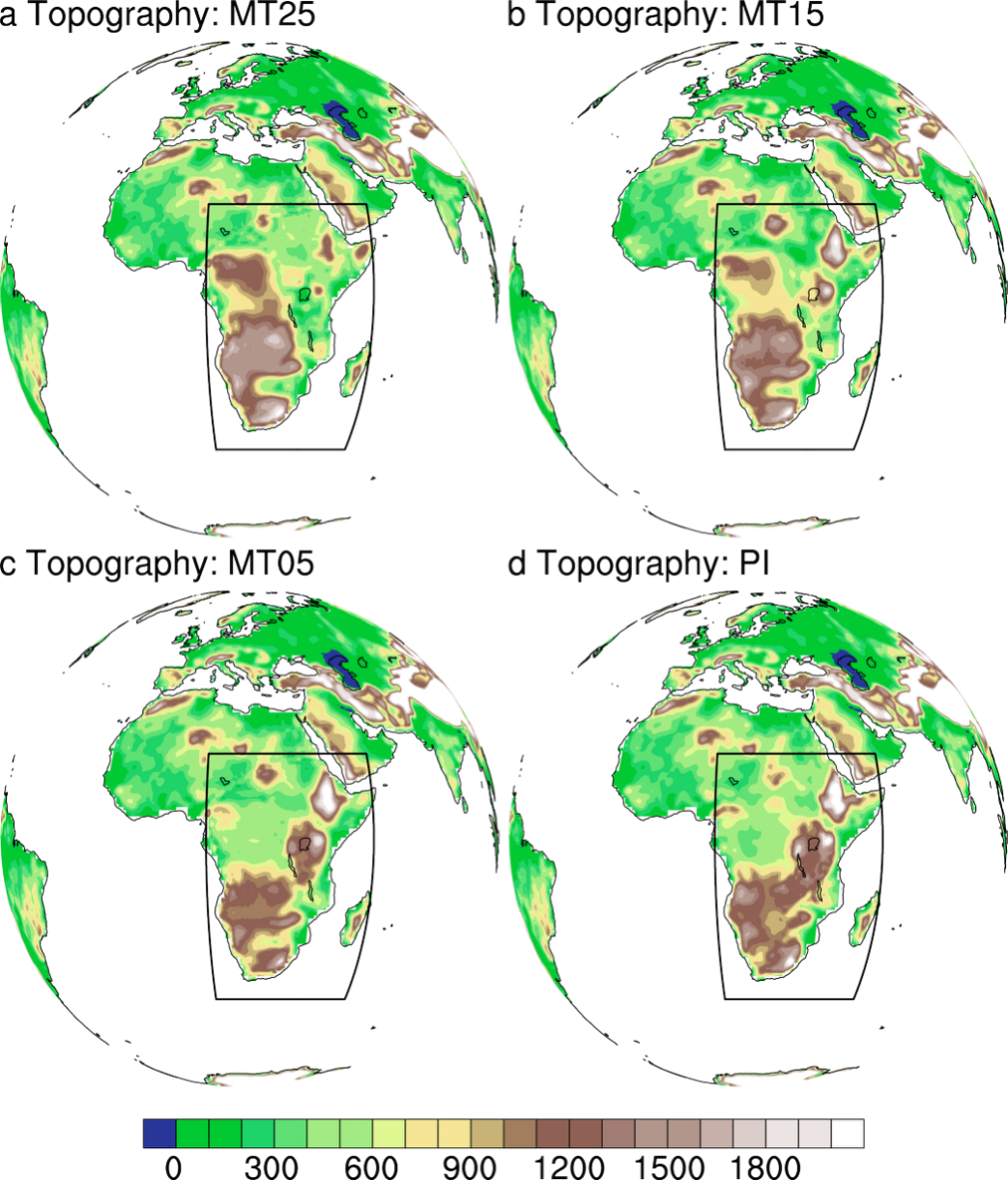


**Supplementary Figure 2 African topography since the Miocene. a** Early Miocene (~25 Myr), **b** Middle Miocene (~15 Myr), **c** Late Miocene (~5 Myr) and **d** modern time slices. Only the African topography is changed based on the paleo-topography map created in ref. ^3^. The solid boxes mark the African region. Units: m.

**
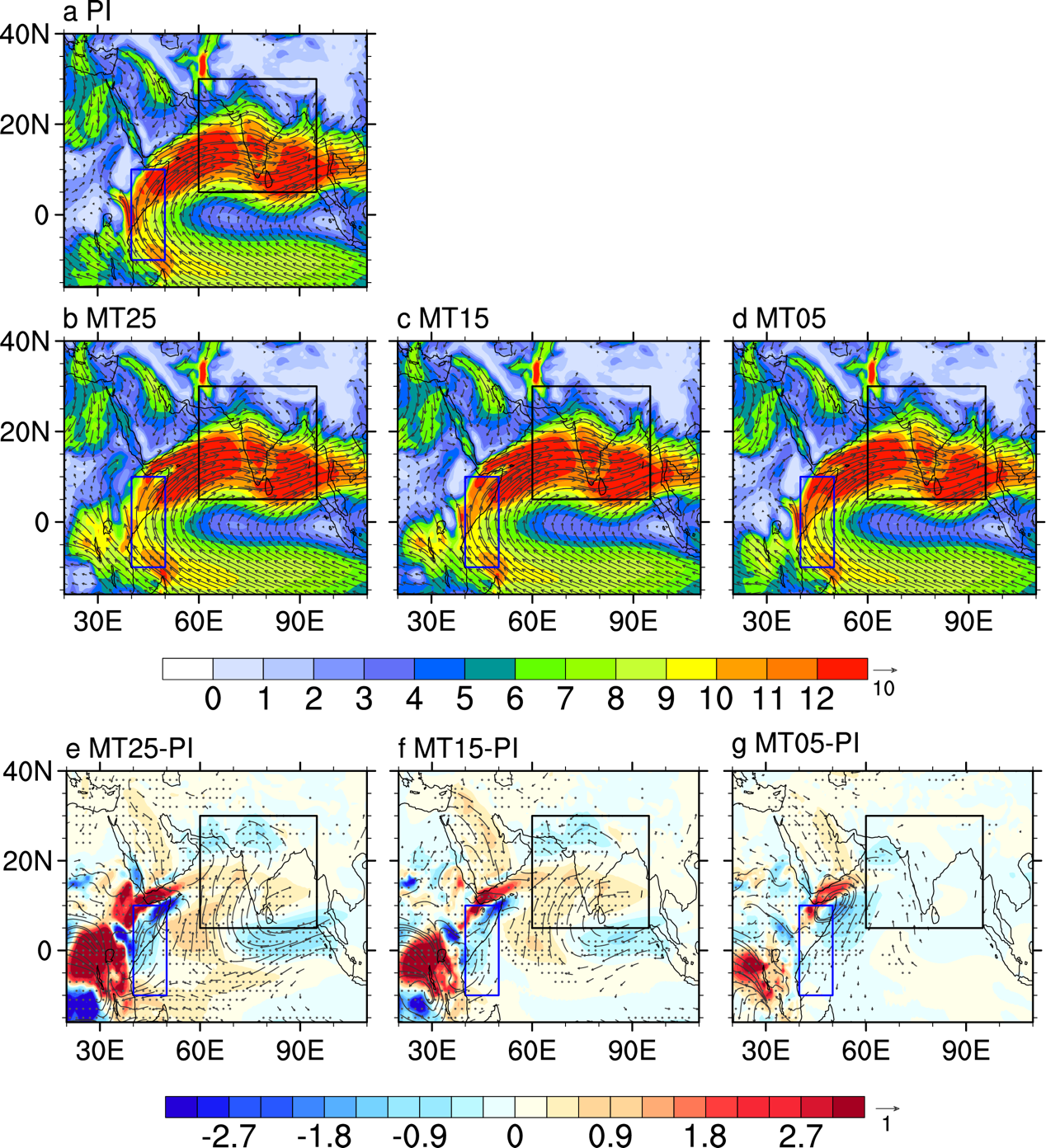
**

**Supplementary Figure 3 Changes in atmospheric circulation due to African topography changes during boreal summer.** Climatological mean 850-hPa winds (vectors, m s^-1^) and its wind speed (shading, m s^-1^) in the **a** pre-industrial, **b** MT25, **c** MT15 and **d** MT05 simulations. Responses to Miocene African topography changes in **e** MT25, **f** MT15 and **g** MT05 simulations compared to the pre-industrial simulation. Black boxes mark the SASM region, and blue boxes mark the cross-equatorial Somali Jet. In **e**-**g**, gray stippling denotes regions in which the changes are significant at the 95% confidence level according to Student’s *t*-test.


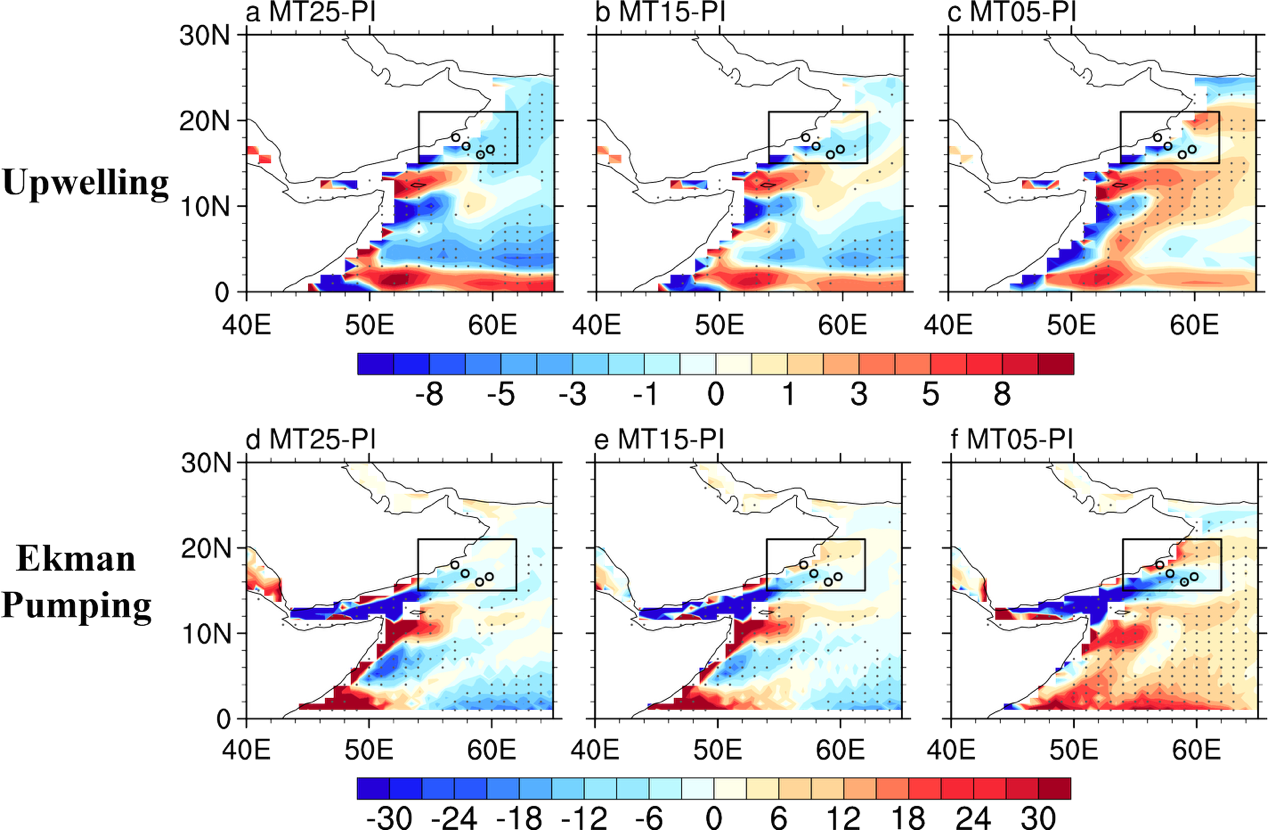


**Supplementary Figure 4 Responses of ocean upwelling over western Arabian Sea to African topography changes during boreal summer**. Changes in vertical velocity of surface ocean (0-100m average, cm day^-1^) in the **a** MT25, **b** MT15 and **c** MT05 simulations compared to pre-industrial simulations. **d**-**f** Same as **a**-**c**, but for the changes in Ekman pumping (see Methods, cm day^-1^). The gray circles in western Arabian Sea denote the locations of the proxy records in Figure 1**i** and **j**. The black boxes denote the western Arabian Sea upwelling region that proxy records located. Gray stippling denotes regions in which the changes are significant at the 95% confidence level according to Student’s *t*-test.


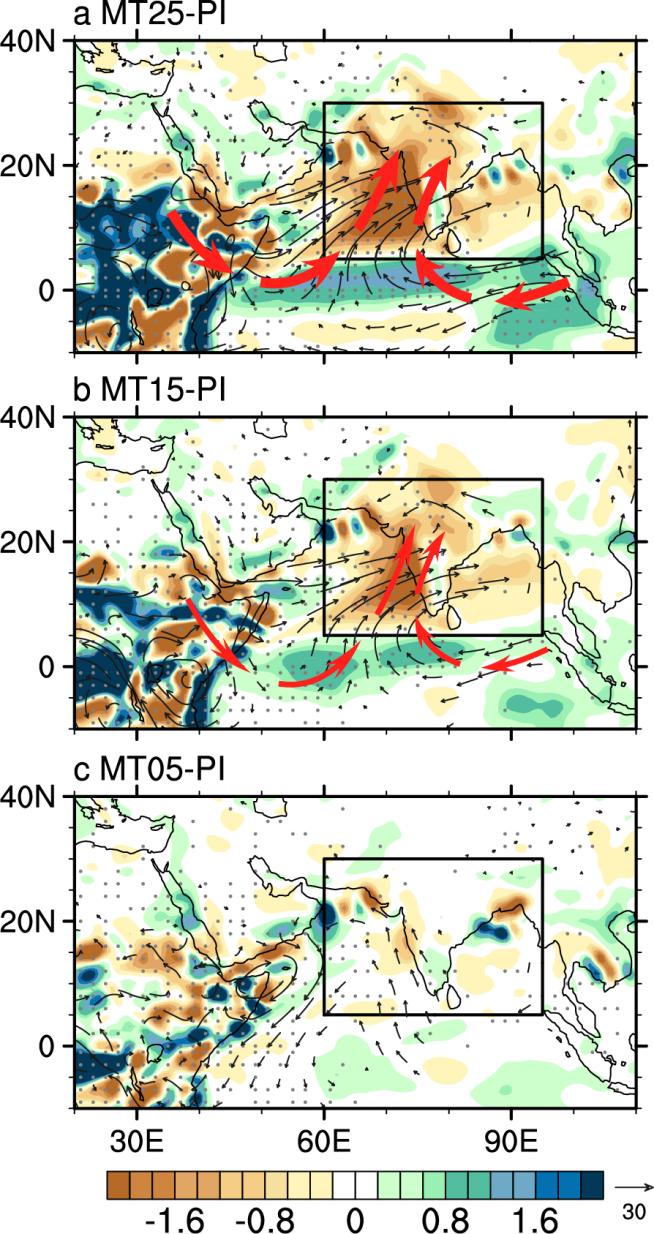


**Supplementary Figure 5** **Changes in moisture transport due to African topography changes during summer.** Changes in vertical integral water vapor flux from 1000 hPa to 10 hPa (vectors, kg m^-1^ s^-1^) and its divergence (shading, mm day^-1^) in the **a** MT25, **b** MT15 and **c** MT05 simulations relative to the pre-industrial simulations. Red bold vectors in **a** and **b** indicate the moisture channel anomalies. In **a**-**c**, gray stippling and vectors denote regions in which the changes are significant at the 95% confidence level according to Student’s *t*-test, and solid boxes mark the SASM region.


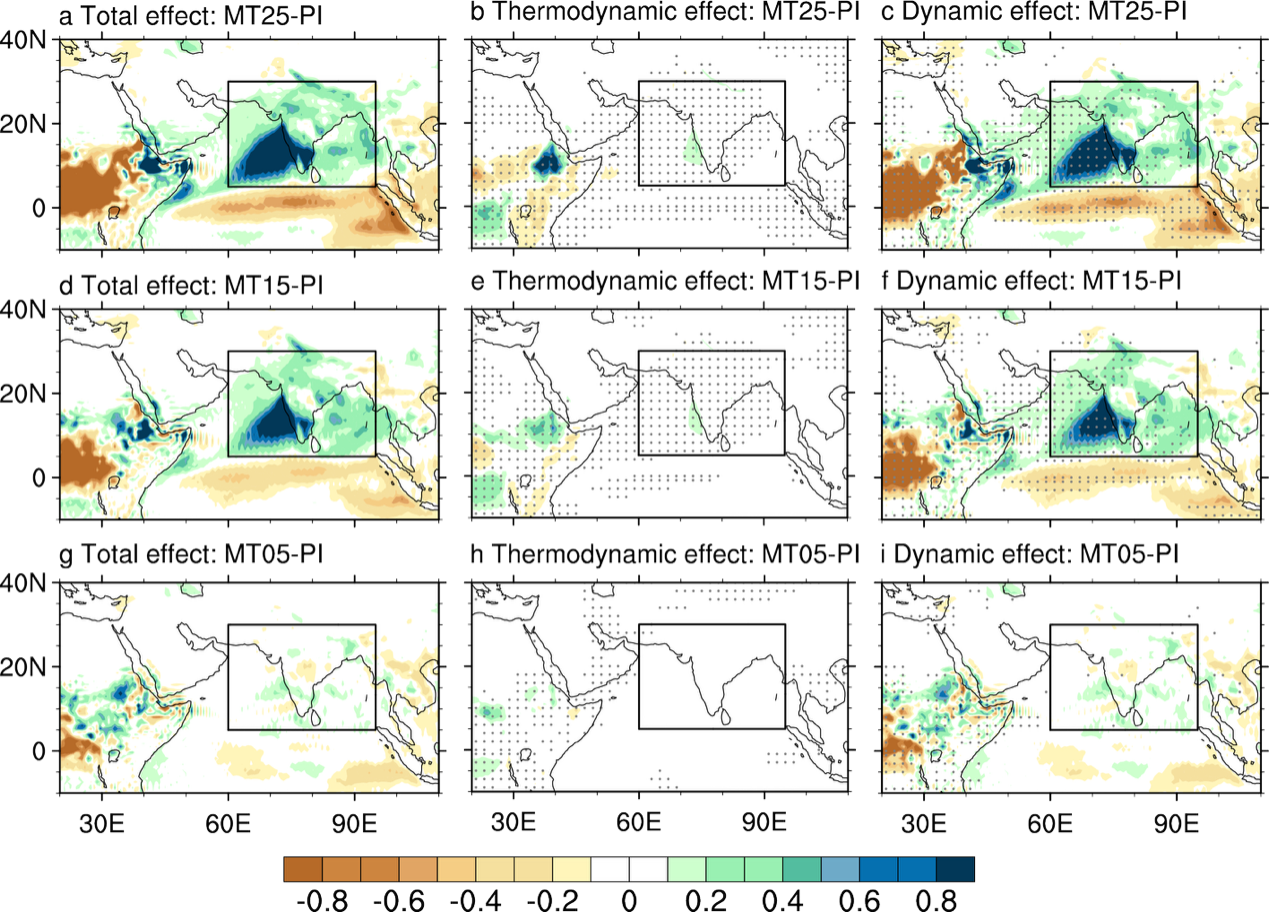


**Supplementary Figure 6 Moisture budget analysis of changes in summer rainfall due to African topography changes. a**, **d**, and **g** Diagnosed changes in rainfall (i.e., the sum of thermodynamic and dynamic terms) in MT25, MT15 and MT05 simulations compared to pre-industrial simulations, respectively. **b**, **e** and **h** Same as **a**, **d**, and **g**, but for the thermodynamic terms. **c**, **f** and **i** Same as **a**, **d**, and **g**, but for the dynamic terms. See Methods for details of the decomposed atmospheric moisture budget. The solid boxes mark the SASM region. Gray stippling in each panel denotes regions in which the changes are significant at the 95% confidence level according to Student’s *t*-test, and solid boxes mark the SASM region. Units: Pa kg m^-2^ s^-1^.


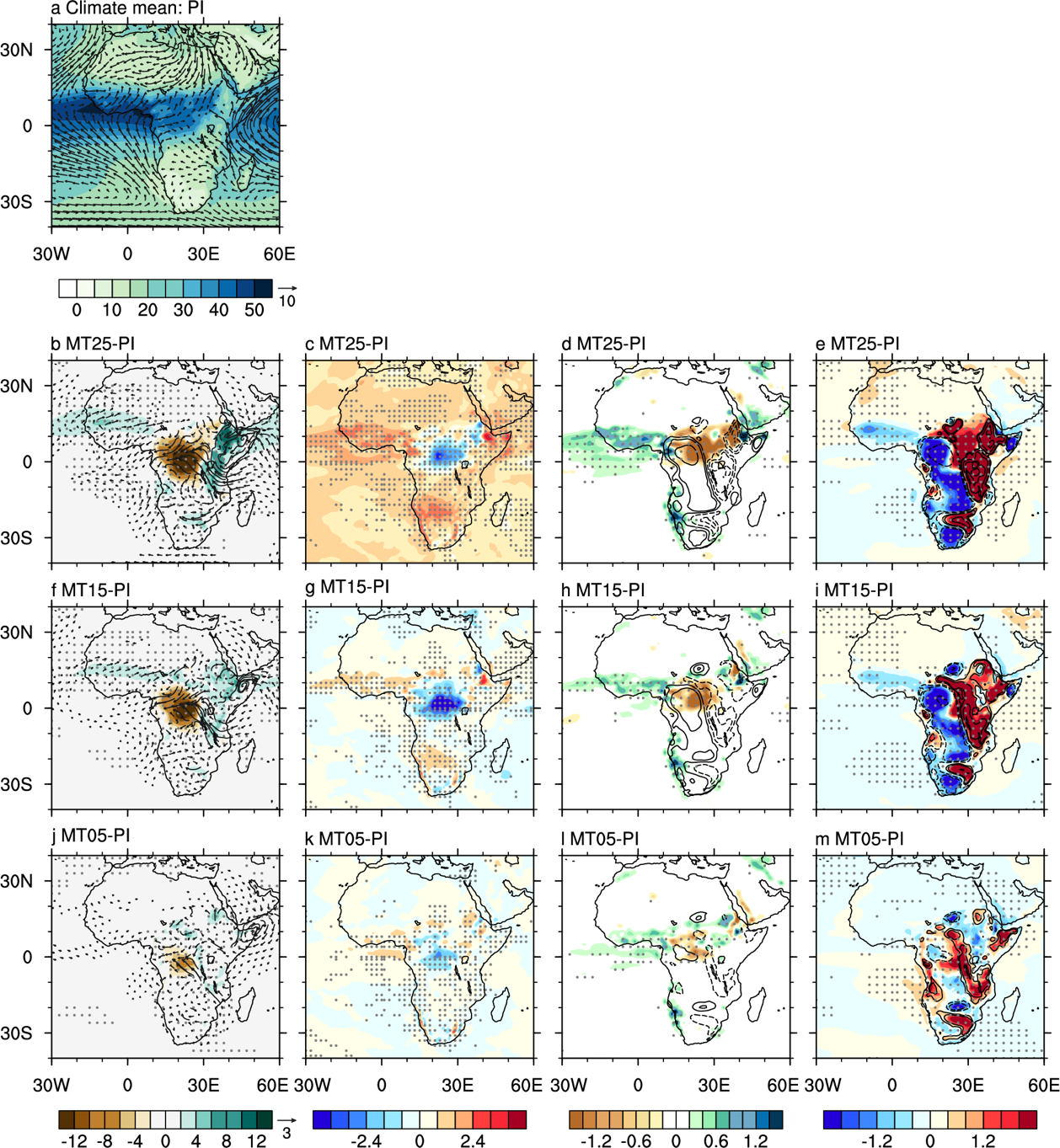


**Supplementary Figure 7** **The impact of African topography changes on decreased rainfall over central Africa during the Miocene. a** Climatological mean precipitable water vapor (shading, kg m^-1^ s^-1^) and horizontal wind (vectors, m s^-1^) at 925 hPa for the pre-industrial simulation. Responses to Miocene African topography changes in **b** MT25, **f** MT15 and **j** MT05 simulations compared to the pre-industrial simulation. **c**, **g**, and **k** Same as **b**, **f**, and **j**, but for the responses of vertical velocity at 500 hPa (-10^-2^ Pa s^-1^; Negative value indicates downward velocity). **d**, **h**, and **l** Same as **b**, **f**, and **j**, but for the responses of moisture condensation (Q_L_; shading, 10^-2^ m^2^ s^-3^; see Methods for details) at 500 hPa, overlaid with topography changes [contours; m; solid (dashed) contours are positive (negative) values] in MT25, MT15 and MT05 simulations compared to the pre-industrial simulation. **e**, **i**, and **m** Same as **b**, **f**, and **j**, but for the responses of surface air temperature (shading, ℃), overlaid with surface pressure changes [contours; hPa; solid (dashed) contours are positive (negative) values] in MT25, MT15 and MT05 simulations compared to the pre-industrial simulation. Gray stippling in **b**-**j** and vectors in **b**, **e** and **h** denote regions in which the changes are significant at the 95% confidence level according to Student’s *t*-test.


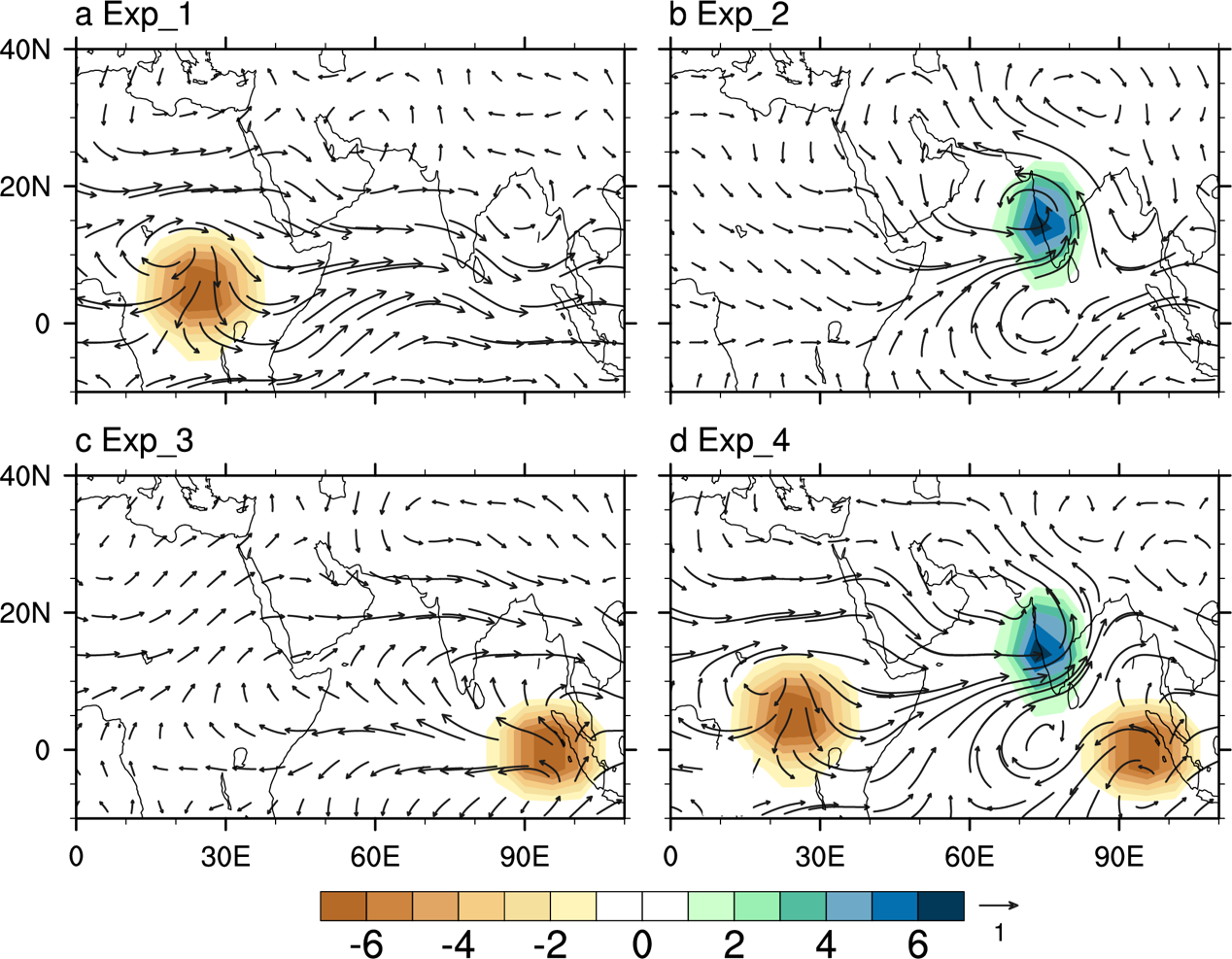


**Supplementary Figure 8 Atmospheric responses to deep cooling/heating in LBM experiments. a** Response of 700-hPa horizontal wind (vectors, m s^-1^) to a prescribed cooling anomaly (shading, K day^-1^) at around 450 hPa over tropical central Africa (see Method for details). **b** and **c** Same as in **a**, but for the cooling over the tropical eastern Indian Ocean and heating over the SASM region. **d** Same as in **a**, but for considering the combined impact of cooling/heating anomaly on atmospheric circulation.


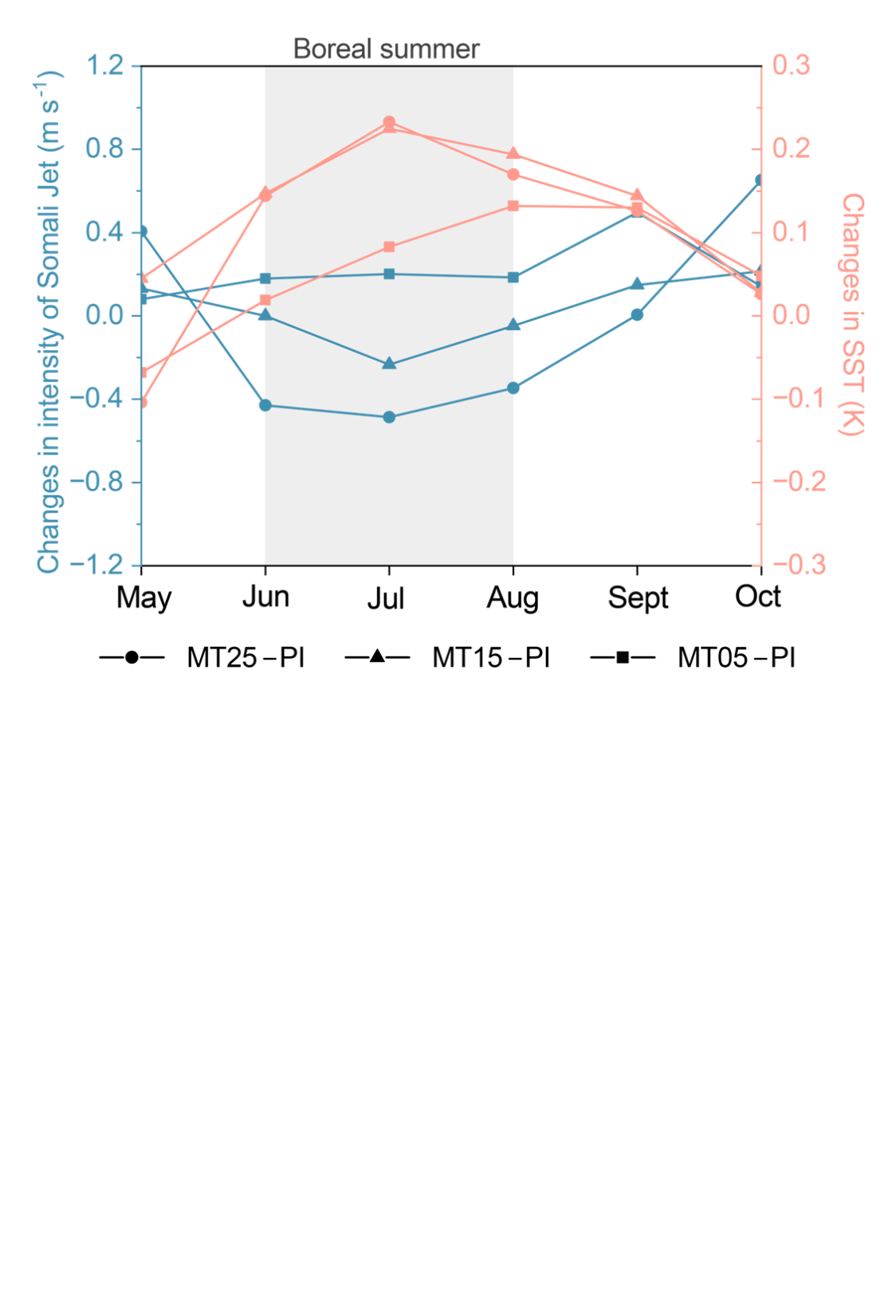


**Supplementary Figure 9 Strong coupling of the intensity of Somali Jet and sea surface temperature (SST) over the western Arabian Sea.** Seasonal variation of changes in intensity of Somali Jet (m s^-1^; see Methods for details) and SST (℃) over tropical western Indian Ocean (10° S-10° N, 40° -55° E). The vertical shaded areas represent the boreal summer (June to August).


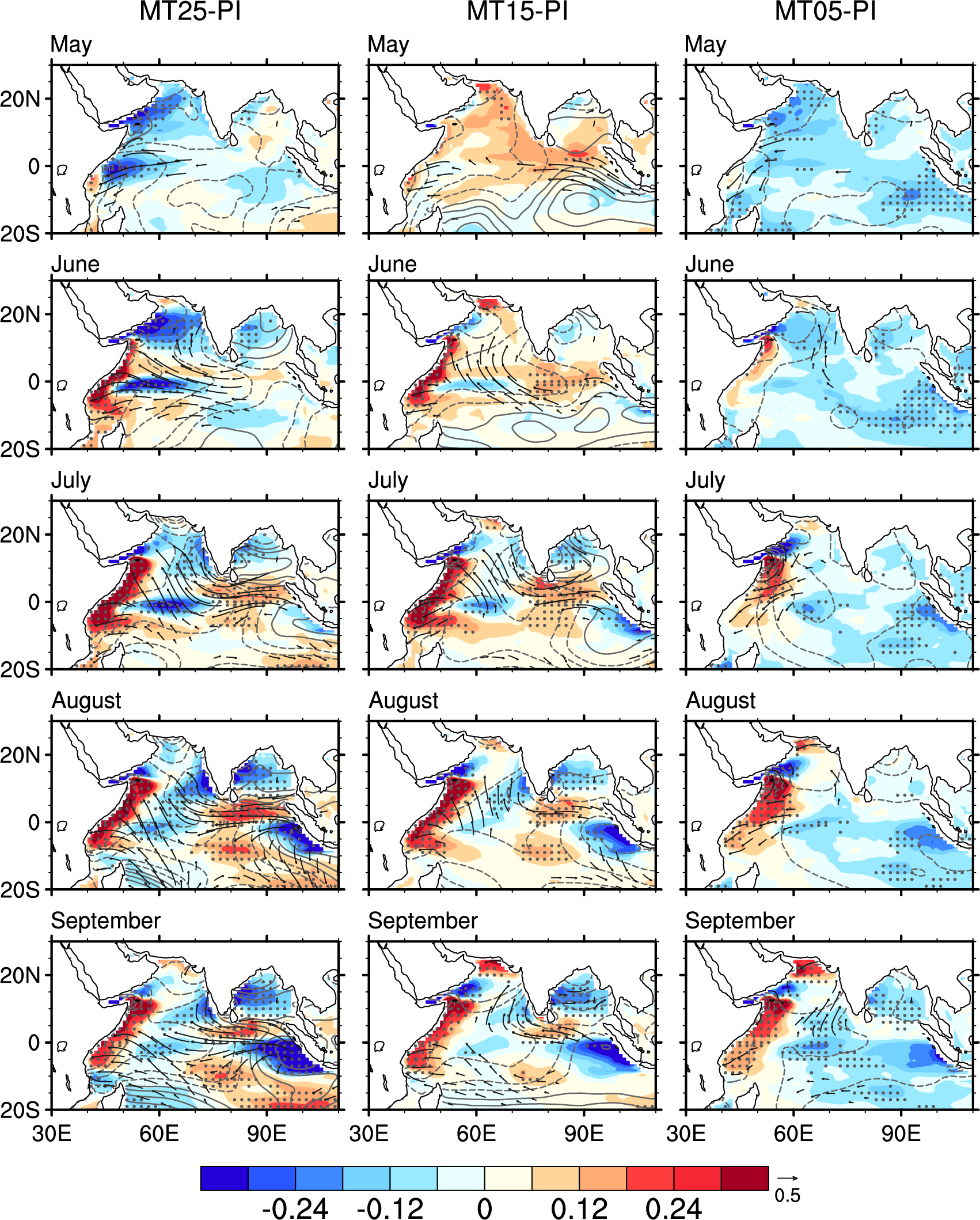


**Supplementary Figure 10 Seasonal variation**. Changes in SST (shading, ℃), sea level pressure [contours; hPa; solid (dashed) lines represent the positive (negative) values], and surface wind (vectors, m s^-1^) from May to September in the MT25 (left column), MT15 (middle column) and MT05 (right column) simulations compared to pre-industrial simulation. Gray stippling and vectors in each panel denote regions in which the changes are significant at the 95% confidence level according to Student’s *t*-test.

**~~
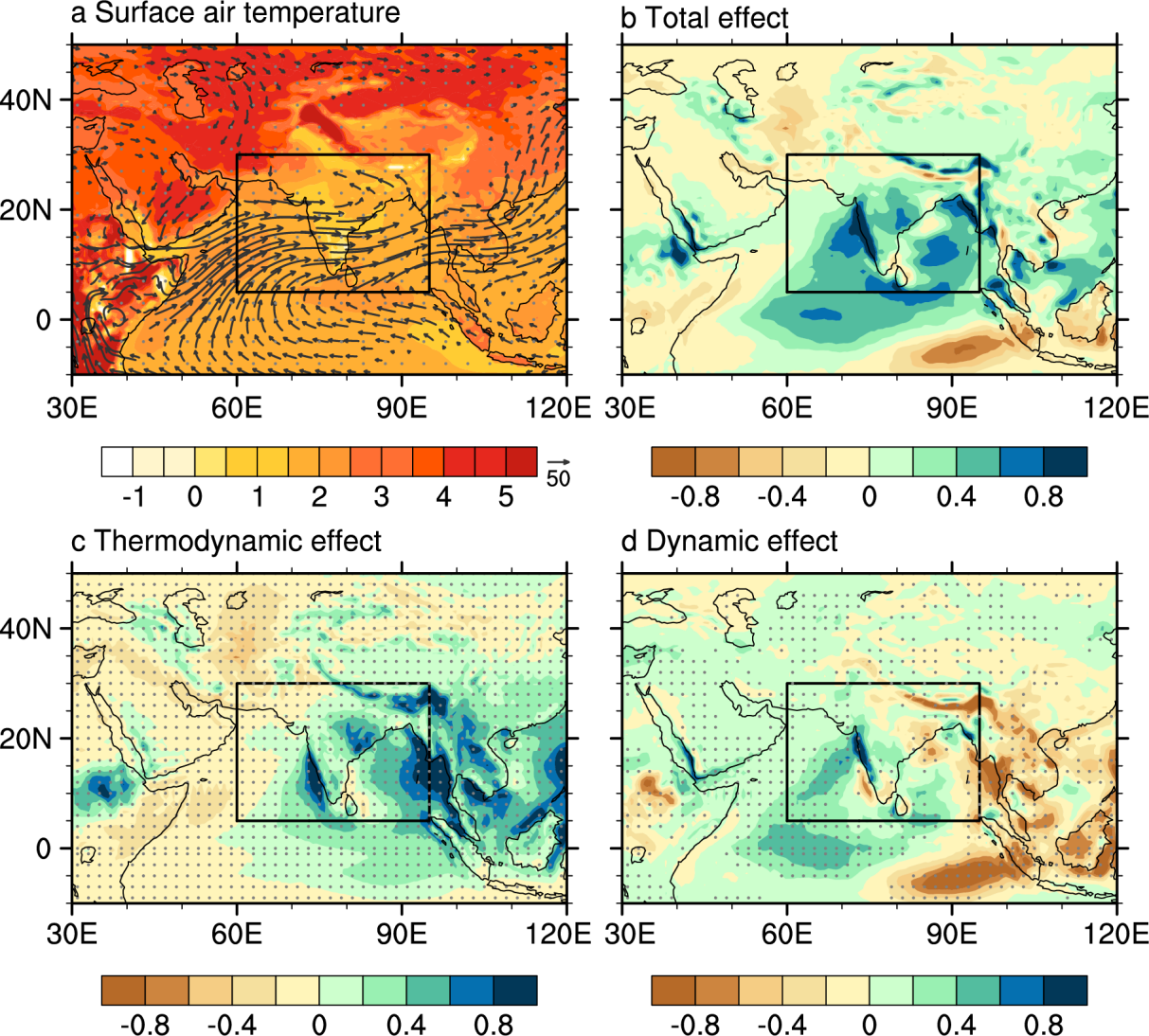
~~**

**Supplementary Figure 11** **Moisture budget analysis of the changes in summer rainfall due to increased CO_2_ concentration. a** Changes in air temperature (shading, ℃) and vertical integral water vapor flux from 1000 hPa to 10 hPa (vectors, kg m^-1^ s^-1^). Changes in **b** diagnosed rainfall (i.e., the sum of thermodynamic and dynamic terms), **c** thermodynamic term, and **d** dynamic term in MC25 simulation compared to MT25 simulation. See Methods for details of the decomposed atmospheric moisture budget. Gray stippling in **a**, **c** and **d**, and vectors in **a** denote regions in which the changes are significant at the 95% confidence level according to Student’s *t*-test. The units in **b**-**d** are Pa kg m^-2^ s^-1^. The solid boxes in each panels mark the SASM region.


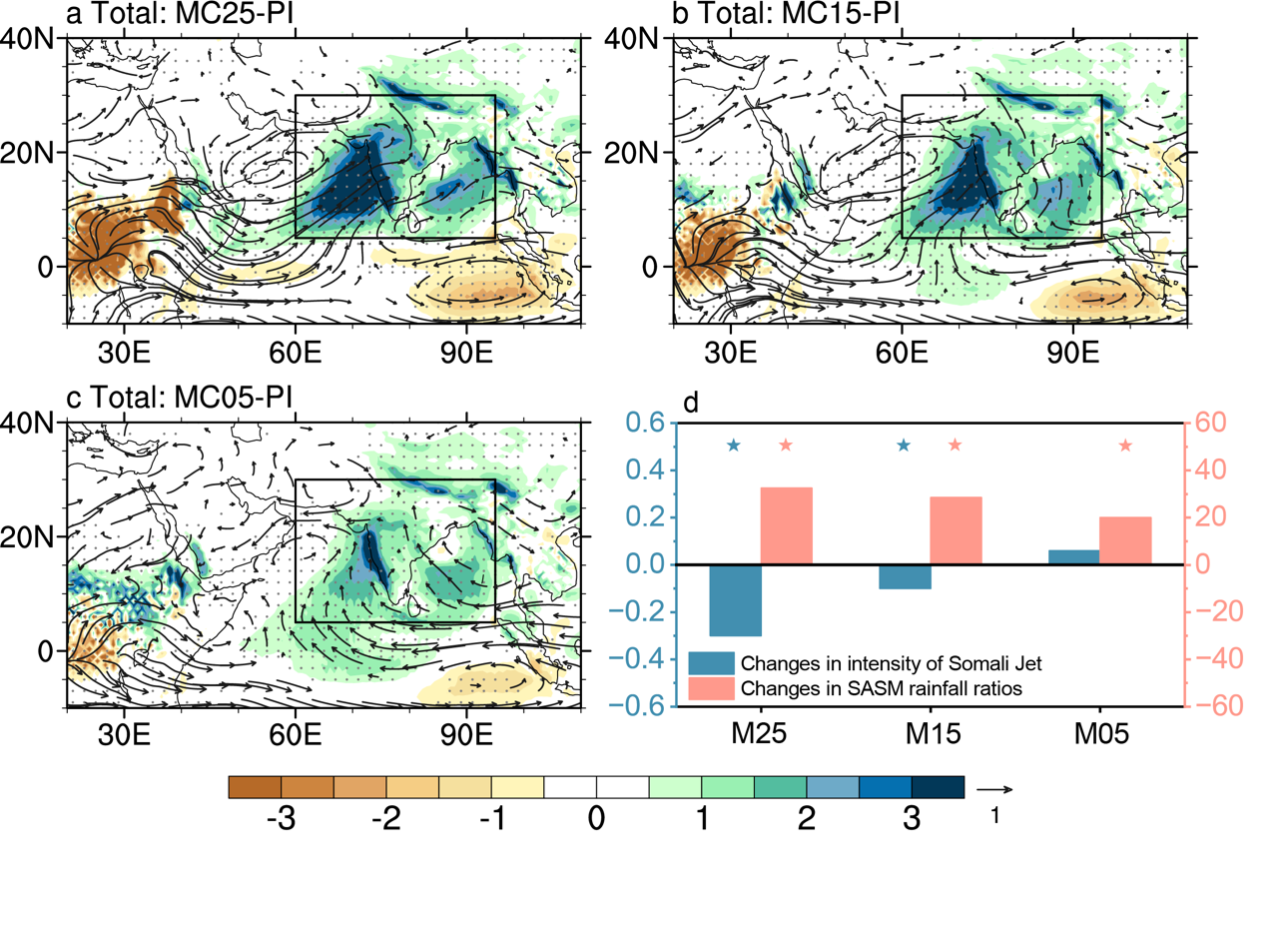


**Supplementary Figure 12** **Changes in SASM rainfall and atmospheric circulation due to combined effects of African topography changes and elevated CO_2_ levels.** Changes in rainfall (shading, mm day^-1^) and 700-hPa wind (vectors, m s^-1^) in the **a** MC25, **b** MC15 and **c** MC05 simulations relative to the pre-industrial simulation. **d** Area-averaged changes in Somali Jet intensity (m s^-1^; see Methods) and ratios of summer rainfall (%) over the SASM region in **a**-**c** due to combined effect of African topography and CO_2_ forcings, with asterisks above the bars denoting changes that are significant at the 95% confidence level according to Student’s *t*-test. In **a**-**c**, gray stippling and vectors denote regions in which the changes are significant at the 95% confidence level according to Student’s *t*-test.


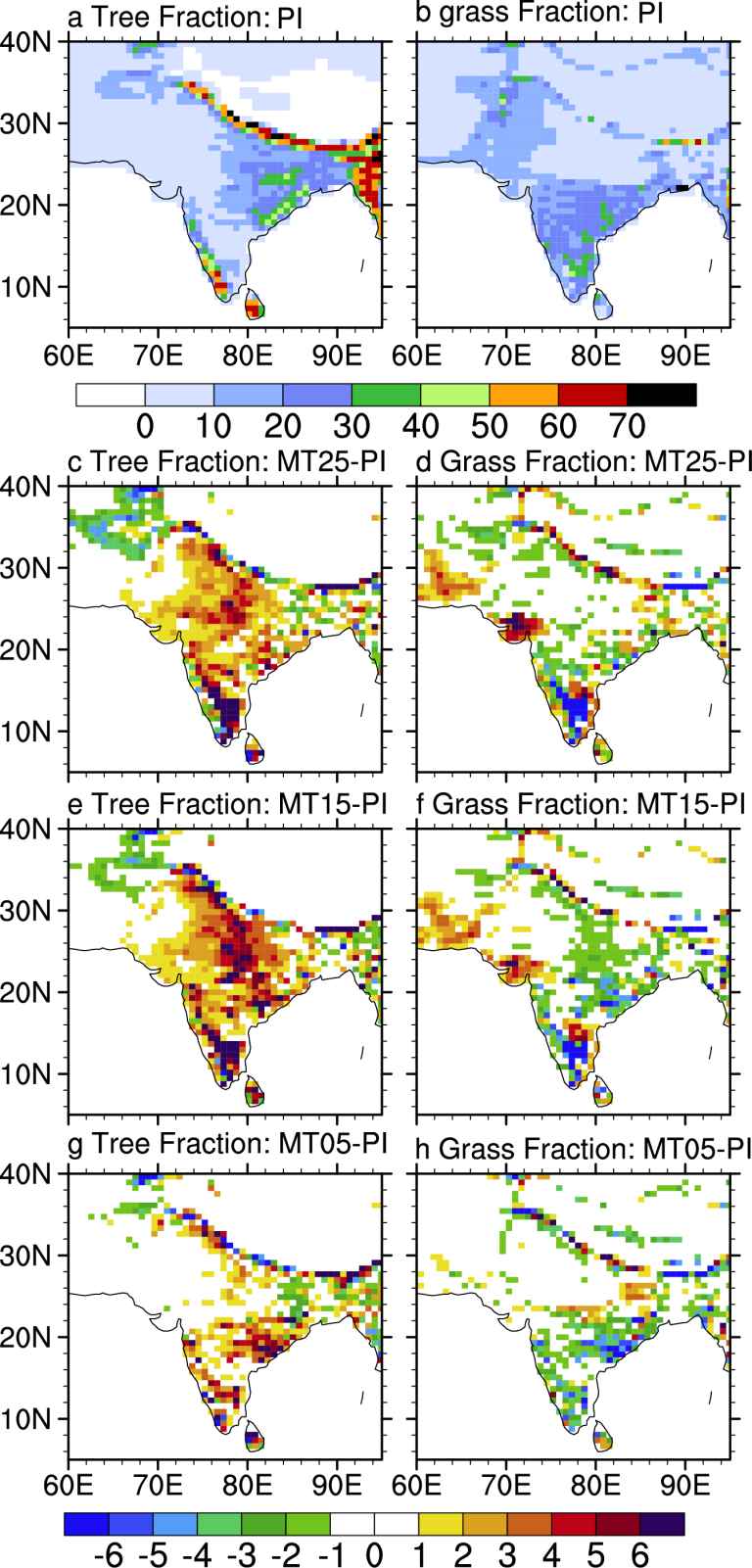


**Supplementary Figure 13** **Changes in vegetation fraction due to African topography changes during summer.** Climatological means of **a** tree fraction and **b** grass fraction in the pre-industrial simulation. Changes in **c** tree fraction and **d** grass fraction in MT25 simulation compared to pre-industrial simulation. **e**-**f** and **g**-**h** Same as **c**-**d**, but for the MT15 and MT05 simulations compared to PI simulation, respectively. Units: %.

**Supplementary References**

1. Adler, R. F. *et al.* The Global Precipitation Climatology Project (GPCP) Monthly Analysis (New Version 2.3) and a Review of 2017 Global Precipitation. *Atmosphere* **9**, 138 (2018).

2. Kanamitsu, M. *et al.* Ncep–doe amip-ii reanalysis (r-2). B. Am. Meteorol. Soc. **83**, 1631-44 (2002)

3. Moucha, R. & Forte, A. M. Changes in African topography driven by mantle convection. *Nat. Geosci.* **4**, 707–712 (2011).
